# A Retrospective Study Comparing Olaparib and Bevacizumab as a Maintenance Therapy for Platinum-Sensitive Recurrent Ovarian Cancer: Impact on Recurrence-Free Survival in Japanese and Asian Populations

**DOI:** 10.3390/cancers15102869

**Published:** 2023-05-22

**Authors:** Kazuho Nakanishi, Masafumi Toyoshima, Yuta Ueno, Shunji Suzuki

**Affiliations:** 1 Department of Obstetrics and Gynecology, Nippon Medical School Chiba Hokusoh Hospital, Chiba 270-1694, Japan; 2 Department of Obstetrics and Gynecology, Nippon Medical School, Tokyo 113-8603, Japan; m-toyoshima@nms.ac.jp (M.T.); yueno@nms.ac.jp (Y.U.); czg83542@mopera.ne.jp (S.S.)

**Keywords:** platinum-sensitive recurrent ovarian cancer, bevacizumab, olaparib, chemotherapy, maintenance therapy

## Abstract

**Simple Summary:**

This study aimed to determine the optimal maintenance therapy and selection criteria for patients with platinum-sensitive recurrent ovarian cancer (PSROC). The study collected retrospective data from 51 patients with PSROC and found that olaparib (Ola) was superior to bevacizumab (Bev) as a maintenance therapy, with a significantly prolonged recurrence-free survival (RFS) of 27 months in the Ola group compared to 9 months in the Bev group. The efficacy of Ola was independent of background factors, such as response to previous chemotherapy and homologous recombination status. These results suggest that Ola is a better treatment option than Bev for patients with PSROC, especially in Japanese and Asian populations.

**Abstract:**

The use of angiogenesis inhibitors and poly ADP-ribose polymerase inhibitors following multi-agent chemotherapy, including platinum-based agents, has become the standard treatment for platinum-sensitive recurrent ovarian cancer (PSROC). However, the optimal maintenance therapy and selection criteria for these patients remain unclear. Thus, this study aimed to optimize the treatment options and selection criteria for patients with PSROC. The clinical data of 51 patients with PSROC admitted to Nippon Medical School Chiba Hokusoh Hospital and Nippon Medical School Hospital were retrospectively collected. The log-rank test was used for the survival analysis, and Cox proportional hazard regression analysis was used for the multivariate survival analysis. Of the 51 patients, 17 received maintenance therapy with bevacizumab (Bev), and 34 received olaparib (Ola). Recurrence-free survival (RFS) was significantly prolonged in the Ola group (27 months; 95% confidence interval (CI), 19–NA months) compared with that in the Bev group (9 months; 95% CI, 5–22 months; *p* = 0.000103). The efficacy of Ola was independent of background factors, including response to previous chemotherapy, homologous recombination status, histological type, or laboratory data. Ola is superior to Bev as PSROC maintenance therapy, especially in Japanese and Asian populations.

## 1. Introduction

Recently, with the advent of biologics and the practical application of genetic testing, there has been a paradigm shift in drug therapy for recurrent ovarian cancer. Molecularly targeted therapies, such as angiogenesis inhibitors, poly ADP-ribose polymerase (PARP) inhibitors, and immune checkpoint inhibitors, have been introduced into clinical practice. With the emergence of these novel agents, treatment selection for recurrent ovarian cancer has been based on the biological characteristics of the disease, including BRCA mutation status and homologous recombination deficiency (HRD). However, most evidence for ovarian cancer accumulated to date has been categorized as “platinum-sensitive recurrence (PSR)” and “platinum-resistant recurrence (PRR)”. Therefore, treatment options for recurrent ovarian cancer are recommended for each of these recurrence patterns, for example, combination therapy with platinum-based agents for PSR. PSR is the time from the end of platinum therapy to recurrence in 6 months or more, whereas PRR is the time from the end of platinum therapy to recurrence in fewer than 6 months [1].

The most recent National Comprehensive Cancer Network guidelines [2] and the Japanese Society of Gynecologic Oncology Guidelines for the treatment of ovarian, fallopian tube, and peritoneal cancer [3] recommend maintenance therapy with either bevacizumab (Bev) or olaparib (Ola) following successful platinum-containing combination therapy. The Japanese Society of Gynecologic Oncology Guidelines recommend the following drug regimens for PSR ovarian cancer (PSROC): multiple-drug regimens, including platinum-based agents; maintenance with Bev in addition to multi-agent chemotherapy; maintenance with Ola or niraparib after response to platinum-containing chemotherapy; and monotherapy with niraparib for patients with HRD and platinum-sensitive relapsed disease who had received at least three prior regimens of chemotherapy. However, a clear recommendation on whether Bev or Ola should be used for PSROC or what selection criteria should be followed remains unclear.

Therefore, this study aimed to optimize the treatment options and selection criteria for PSROC by retrospectively examining the clinical data of patients with PSROC at the Nippon Medical School Chiba Hokusoh Hospital, Chiba, Japan, and the Nippon Medical School Hospital, Tokyo, Japan.

## 2. Materials and Methods

### 2.1. Ethics Approval

Patient consent to participate in the study was obtained in advance in writing, and a summary of the study was published in an opt-out manner according to our institution’s standards. Ethics approval was obtained from the Ethics Committee of Nippon Medical School Chiba Hokusoh Hospital (approval number: 874-2).

### 2.2. Patient History

Between 1 January 2015, and 30 July 2022, 58 patients were diagnosed with PSROC at Nippon Medical School Chiba Hokusoh Hospital and Nippon Medical School Hospital, Tokyo, Japan. Of these 58 patients, 17 received platinum-based chemotherapy plus Bev followed by single-agent maintenance therapy with Bev, and 34 received platinum-based chemotherapy followed by maintenance therapy with Ola. Three patients stopped treatment during maintenance therapy, and one patient died of another disease. In addition, one patient on Bev received maintenance therapy with niraparib, one refused maintenance therapy owing to intolerable adverse events, and one stopped active treatment because she wanted the best supportive care. All the remaining patients were included in the study, and none had contraindications that would have precluded administration of the two drugs. Overall, 51 patients diagnosed with PSROC and treated with Bev or Ola were included. The observation period did not include the chemotherapy period; only the Bev maintenance period after multi-agent chemotherapy with Bev and the Ola maintenance period after the completion of chemotherapy were included. The Bev and Ola treatment groups included five patients who received both Bev and Ola at different times (three patients received Bev first, and two patients received Ola first).

### 2.3. Study Design

The clinical data of 51 patients with platinum-sensitive ovarian cancer treated at Nippon Medical School Chiba Hokusoh Hospital and Nippon Medical School Hospital between 2015 and 2022 were retrospectively studied. The patients were divided into the Bev and Ola groups, and the attending physician determined their choice of a maintenance drug. Olaparib was approved in Japan on 19 January 2018 for maintenance therapy in PSROC. Thus, Bev was more frequently selected before the approval date, and Ola was more frequently selected after that date. The age, histological type, and number of treatment regimens to relapse for all patients are shown in Table 1. To detect treatment response bias due to patient characteristics, we additionally examined the NLPN score [4], neutrophil–lymphocyte ratio (NLR) [5,6], and systemic immune-inflammation index (SII) [7], which are predictors of response to treatment for ovarian cancer. The primary endpoint was recurrence-free survival (RFS), defined as the interval between the start of treatment and the first radiologically proven disease progression or death, whichever occurred first. The secondary endpoint was overall survival (OS), defined as the period from the start of treatment to the last confirmed date of survival before death.

### 2.4. Blood Sample Analysis

Blood samples were collected during pre-operative testing, postoperative chemotherapy, and post-treatment follow-up. The collected samples were analyzed to determine the white blood cell, neutrophil, and lymphocyte counts using a multi-item automatic blood cell analyzer (XE-2100; Sysmex, Kobe, Japan). A chemical autoanalyzer (Hitachi 7700; Hitachi, Ibaraki, Japan) was used to determine serum lactate dehydrogenase (LDH) and C-reactive protein (CRP) levels. LDH levels were analyzed using an LDH kit (L-type LD J, FUJIFILM Wako Pure Chemical Corporation, Osaka, Japan). CRP levels were analyzed using N-assay LA CRP-S D-type assay (Nittobo Medical, Tokyo, Japan) in accordance with the manufacturer’s protocols. Serum cancer antigen (CA) 125 levels were determined using an ARCHITECT i2000SR immunoassay analyzer (Abbott, Chicago, IL, USA).

Biopsy or surgical specimens were used to detect HRD by assessing the genomic instability status of genomic DNA and determining BRCA1 or BRCA2 gene variants in the genomic DNA extracted from tumor tissue using myChoice^®^ CDx (Myriad Genetics, Inc., Salt Lake City, UT, USA). For patients with somatic BRCA mutations, we recommended the confirmation of BRCA mutations in the germline using BRCAnalysis^®^ (Myriad Genetics, Inc., Salt Lake City, UT, USA) or single-site testing.

### 2.5. Histopathology

Surgical tissue specimens obtained from the debulking procedure were stained with hematoxylin and eosin to determine the histologic cancer type. Serous cancer specimens were analyzed for p53 overexpression by immunostaining with anti-p53 (DO-7) antibody (GeneTex, Inc., Irvine, CA, USA). Moreover, we used the revised Response Evaluation Criteria in Solid Tumors (RECIST) guidelines (version 1.1) to assess the therapeutic effect on patients after platinum-based treatment if they had a complete response (CR) or partial response (PR).

### 2.6. Statistical Analysis

Differences in age; white blood cell, neutrophil, lymphocyte, and platelet counts; and NLR, SII, NLPN, CA125, and LDH levels were analyzed using *t*-tests. The Mann–Whitney U test was used to analyze the observation period (from the start of maintenance treatment to the last confirmed date of survival) for both groups. The stage, histology, homologous recombination status, therapeutic effect, and the number of previous regimens in both groups were analyzed using Fisher’s exact test. Survival analysis was performed using the log-rank test. All tests were two-tailed, and *p* < 0.05 was considered statistically significant. All statistical analyses were performed using EZR (Saitama Medical Center, Jichi Medical University, Saitama, Japan), a graphical user interface for R designed to include statistical functions (R Foundation for Statistical Computing, Vienna, Austria) [8].

## 3. Results

Of the 17 patients in the Bev group and the 34 patients in the Ola group, 15 (88.2%) and 14 (41.2%) patients relapsed, respectively. The platinum-based treatment regimens used in the 51 patients included paclitaxel (PTX) (175 mg/m^2^ on day 1) + carboplatin (CBDCA) (area under the concentration-time curve [AUC] = 5–6 on day 1) ± Bev (15 mg/kg) every 21 days; PTX (80 mg/m^2^ on days 1, 8, and 15) + CBDCA (AUC = 6 on day 1) every 21 days; gemcitabine (1000 mg/m^2^ on days 1 and 8) + CBDCA (AUC = 4 on day 1) every 21 days; irinotecan (60 mg/m^2^ on days 1, 8, and 15) + cisplatin (60 mg/m^2^ on day 1) every 21 days; and doxorubicin (30 mg/m^2^ on day 1) + CBDCA (AUC = 5 on day 1) ± Bev (15 mg/kg *) every 28 days. These treatments lasted for 2 to 4, 4, 4, 4, and 6 months, respectively (* insurance approval in Japan allows this regimen to be used only at a dose of 15 mg/kg every 4 weeks).

Their age, stage at initial treatment, histological type (high-grade serous carcinoma [HGSC] or non-HGSC), homologous recombination status (i.e., HRD, homologous recombination-proficient [HRP], or unknown homologous recombination status) and response after platinum-containing chemotherapy (CR or PR) based on the RECIST guidelines version 1.1 [9], the number of chemotherapy regimens before the start of Bev or Ola treatment, the total white blood cell count (*p* = 0.134), the neutrophil count ratio (*p* = 0.967), the lymphocyte count ratio (*p* = 0.822), the platelet count (*p* = 0.108), the serum CA125 level (*p* = 0.905), the LDH level (*p* = 0.728), the serum CRP level (*p* = 0.727), the NLPN score (*p* = 0.751), NLR (*p* = 0.743), and SII (*p* = 0.183) at the start of therapy did not differ (Table 1).

**Figure 1:**
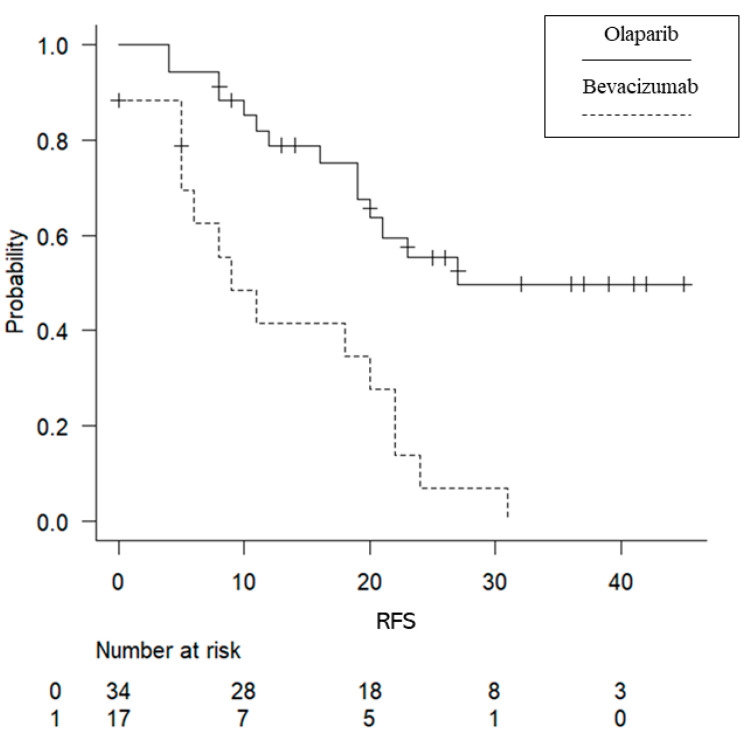
Kaplan–Meier curves for recurrence-free survival (RFS). Patients were grouped into the Bev and Ola groups. The Ola group had a significantly longer RFS.

**Figure 2:**
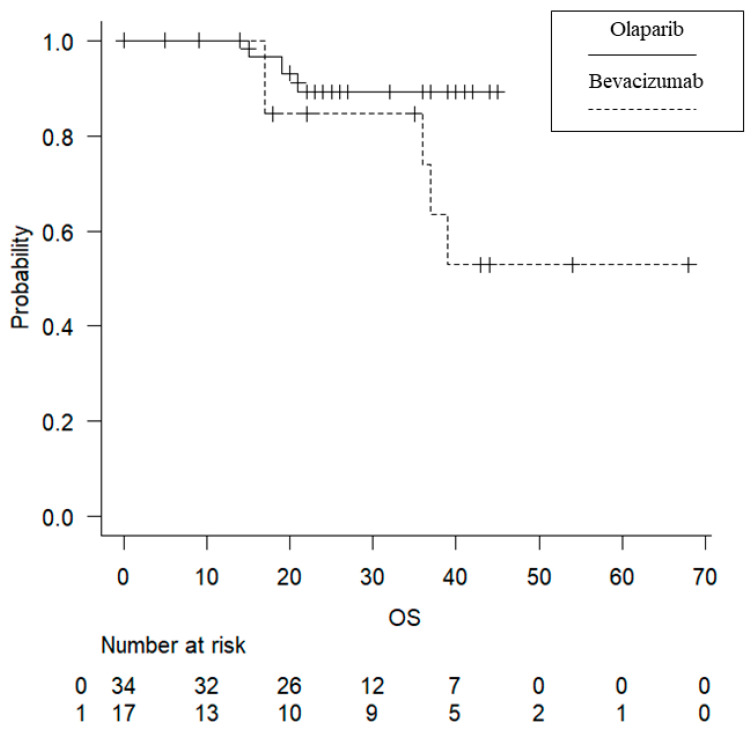
Kaplan–Meier curves for overall survival. No significant difference between the Bev and Ola groups. OS, overall survival.

No significant difference in the observation period (from the start of maintenance treatment to the last confirmed date of survival) was found between the two groups (Ola group, 20 months; Bev group, 17 months; *p* = 0.865). RFS was significantly longer in the Ola group (median progression-free survival: 27 months in the Ola group [95% confidence interval (CI), 19–NA months] and 9 months in the Bev group [95% CI, 5–22 months]; *p* = 0.000103) (Figure 1). No significant difference in OS was found between the two groups (median OS: Ola group, 34 months [95% CI, NA–NA]; Bev group, 17 months [95% CI, 36–NA], *p* = 0.0953) (Figure 2).

As a secondary exploration, we performed a univariate analysis of the RFS by adding differences in maintenance therapy (i.e., Bev or Ola), HRD status, BRCA status, histopathology type, the best treatment response to the last chemotherapy (i.e., CR or PR), and laboratory abnormalities (i.e., CA125 [>35 IU/mL], LDH [>222 IU/mL], CRP levels [>0.1 mg/dL], NLPN [>7.51], NLR [>3], as well as SII [>730]) at relapse, which has been reported as clinical biomarkers of treatment response to PSROC therapy. No significant differences in any of these indices were found between the two groups (Table 2).

## 4. Discussion

Our findings demonstrate that Ola prolongs RFS when compared to Bev as a maintenance therapy for PSROC. The univariate analysis of the RFS showed no significant differences in the previously reported clinical biomarkers (differences in histology or HRD status, BRCA status, best treatment response to chemotherapy, CA125, LDH, CRP, NLPN score, NLR, and SII). Significant differences were found only in the maintenance therapy drugs. In our study, many cases of unknown HRD status were recorded among the participants (64.7%). This was because myChoice^®^ CDx was approved for insurance in Japan only on 9 September 2020, and many patients were not tested at the time of their first diagnosis. The median RFS of the patients included in this study was longer than that of the BRCAm population in the SOLO2 clinical trial because they were likely to benefit from Ola treatment. Although the use of HRD testing for selecting maintenance therapy is controversial, our results suggest the existence of a population that is more responsive to treatment. The maintenance therapy options for PSROC have been expanded by the results of the OCEANS [10] and GOG-0213 [11] clinical trials for Bev, Study 19 [12], and SOLO2 [13] clinical trials for Ola, and NOVA [14] for niraparib. Recently, several meta-analyses have reported that maintenance therapy with the angiogenesis inhibitor Bev and the PARP inhibitors Ola, niraparib, or rucaparib significantly prolongs RFS and post-progression efficacy outcomes versus the placebo [15,16].

However, only a few studies directly compared individual therapies. To date, the only studies on maintenance therapy for recurrent platinum-sensitive ovarian cancer that specifically compared Ola and Bev include the KGOG analysis of *BRCA*-mutated populations (PubMed search [ovarian cancer] AND [bevacizumab] AND [olaparib]) [17]. The KGOG report indicated that Ola might provide more clinical benefits than Bev. However, only BRCA mutation cases benefited from Ola in the analysis population (median RFS for Ola vs. Bev: 22.2 vs. 17.4 months; *p* = 0.057), which supports the plausibility of our results.

The median RFS of the patients included in this study was 27 months in the Ola group and 9 months in the Bev group. The RFS in the Ola group was longer than those reported in Study 19 (8.4 months; 95% CI, 7.4–11.5 months), which included all-comers, and the SOLO2 trial (19.1 months; 95% CI, 16.3–25.7 months), which analyzed a population of patients with *BRCA* mutations. Although the patient population in the Ola group included many cases of unknown HR status, the distribution of histopathologic types in this study was approximately 60% (29 patients) HGSC, and the response rate to chemotherapy was 44% (26 patients) PR, making it less likely than other studies to include many HRD cases or super-responders, such as patients with BRCA mutations. Studies from Japan and the Asian population reported that the duration of response to Ola might be longer than that observed in previous studies on populations restricted to Japanese and Asian patients, such as those by Yoshihama et al. (15.3 months; 95% CI, 9.0–21.6 months) [18] and Gao et al. (16.1 months; 95% CI, 13.3–18.3 months) [19]. Therefore, ethnic specificity may be a factor in the longer duration of Ola response in the target population in our multicenter analysis.

However, our Bev group had a shorter RFS than that reported in the OCEANS (12.4 months; 95% CI, 11.4–12.7 months) and GOG-0213 (13.8 months; 95% CI, 13.0–14.7 months) studies. The approximately 3–4 months shorter RFS observed in our Bev arm than that reported in the OCEANS or GOG-0213 studies could be because our analysis of the RFS of Ola and Bev alone during maintenance therapy excluded the chemotherapy period. In other words, our groups had a shorter RFS of approximately 4–5 months with chemotherapy than those of the OCEANS or GOG-0213 trials. Therefore, the actual RFS in the Bev arm in this study was comparable to that reported in the OCEANS and GOG-0213 studies.

A subgroup analysis of GOG-218 with Bev showed that the BRCA1/2 group lived 1–3 years longer than the non-BRCA group, indicating that Bev may be effective for some reason in the BRCA mutation group [20]. Therefore, at this point, the idea of not using Bev because of BRCAness may limit treatment options.

As a secondary analysis, we also examined abnormal CA125, LDH, and CRP levels, NLPN score, NLR, and SII, which have been reported as predictors of treatment response in ovarian cancer. We found no difference in the respective values of these parameters between the Bev and Ola groups (*p* = 0.131–0.972). Furthermore, no differences in the pathological stage at initial presentation, degree of response to the immediately preceding chemotherapy, or the number of prior treatments were found between the patients who received Bev and Ola. Therefore, no specific patient population with PSROC is likely to benefit more from Ola than Bev. Although this is a retrospective study on a small number of patients and selection bias cannot be excluded, the development of an Ola monotherapy or a Bev + Ola regimen may be considered rather than maintenance treatment using Bev monotherapy.

The choice of maintenance therapy in the HRP population is even more difficult than that in the HRD population, but because of the large number of patients with unknown HRD in this study, we were unable to demonstrate a difference in efficacy for HRP patients with different maintenance agents.

The rationale for maintenance therapy for recurrent ovarian cancer in the HRP population is based on Bev treatment for subjects without HRD in the OCEANS and GOG-218, Ola for subjects without HR status in Study-19, or niraparib for the HRP population in NOVA. All these studies have demonstrated the efficacy of maintenance therapy compared to that of placebo. Although it remains to be determined whether Ola or Bev should be administered strictly to the HRP population, maintenance therapy should still be administered in the HRP population as part of recurrent ovarian cancer treatment. However, no trials have been conducted in the HRP population to determine which agent is more effective. This is an important topic for future studies on recurrent ovarian cancer, and a large prospective study is expected.

Our results suggest that treatment strategies for PSROC may involve simpler drug selection than considering individual treatments by previously established clinical biomarkers. However, our study had several limitations. First, selection bias and other issues cannot be ruled out because of the retrospective nature of the study. Second, the sample size was limited, and the observation period was short. Third, subgroup analyses based on gene mutations and cumulative dose were not performed. Finally, quality of life, toxicity, adverse events, and cost-effectiveness were not compared. Such information may aid decision-making in managing treatment options owing to BRCA mutations and PSROC. Hence, future prospective, large, multi-racial studies are warranted.

## 5. Conclusions

Our findings suggest that Ola is superior to Bev as a maintenance therapy for PSROC. Furthermore, the efficacy of Ola is independent of background factors, such as response to prior chemotherapy, HRD status, histologic type, and clinical data, especially in the Japanese and Asian populations.

## Figures and Tables

**Table 1:** Clinical background of patients in the Bev and Ola groups.

		Bevacizumab	Olaparib	*p* Value
Patient no.		17	34	
Age		59.0 [40–68]	60.50 [38–76]	0.258
FIGO stage	1	2 [11.8]	4 [11.8]	0.702
	2	0 [0.0]	3 [8.8]	
	3	11 [64.7]	17 [50.0]	
	4	4 [23.5]	10 [29.4]	
HGSC		8 [47.1]	21 [61.8]	0.377
Non-HGSC		9 [52.9]	13 [38.2]	
BRCAm		2 [11.8]	3 [8.8]	0.654
BRCAwt		5 [29.4]	7 [20.6]	
Unknown BRCA status		10 [58.8]	24 [70.6]	
HRD		2 [11.8]	4 [11.8]	0.365
HRP		6 [35.3]	6 [17.6]	
Unknown HR status		9 [52.9]	24 [70.6]	
CR		6 [35.3]	19 [55.9]	0.237
PR		11 [64.7]	15 [44.1]	
Previous chemotherapy (No.)	0	1 [5.9]	2 [5.9]	0.429
	1	9 [52.9]	14 [41.2]	
	2	3 [17.6]	9 [26.5]	
	3	3 [17.6]	3 [8.8]	
	4	0 [0.0]	4 [11.8]	
	5	0 [0.0]	2 [5.9]	
	7	1 [5.9]	0 [0.0]	
White blood cell (/μL)		4900 [3300, 6900]	5550 [3300, 10,800]	0.134
Neutrophil (%)		64.8 [36.6, 76.4]	63.5 [36.6, 76.1]	0.967
Lymphocyte (%)		29.0 [10.7, 54.3]	29.3 [15.0, 54.3]	0.822
Platelet (104/μL)		19.4 [11.0, 31.1]	22.6 [12.3, 38.5]	0.108
CA125 (IU/mL)		64.1 [6.2, 34,000.0]	75.8 [6.8, 1857.6]	0.905
LDH (IU/L)		189.0 [155.0, 304.0]	189.0 [118.0, 326.0]	0.728
CRP (mg/dL)		0.14 [0.05, 3.06]	0.13 [0.03, 3.05]	0.727
NLPN score		3.3 [0.0, 16.3]	3.0 [0.0, 20.9]	0.751
NLR		2.3 [0.7, 7.0]	2.1 [0.7, 5.1]	0.743
SII		42.4 [10.7, 160.1]	48.0 [10.7, 164.1]	0.183

Percentages are in parentheses; lower and upper limits are in square brackets. FIGO, International Federation of Gynecology and Obstetrics; HGSC, high-grade serous carcinoma; wt, wild type; m, mutation; HRP, homologous recombination-proficient; HRD, homologous recombination deficient; HR, homologous recombination; CR, complete response; PR, partial response; CA, cancer antigen; LDH, lactate dehydrogenase; CRP, C-reactive protein; NLR, neutrophil–lymphocyte ratio; SII, systemic inflammatory index; NLPN score, neutrophil–lymphocyte ratio at recurrence × previous number of regimens.

**Table 2:** Univariate analysis of recurrence-free survival (log-rank test).

Variables	*n*	Median Survival	*p* Value
HRD	6	31 (10–NA)	0.617
non-HRD	45	21 (16–27)	
BRCAm	5	31 (10–NA)	0.706
BRCAwt or unknown	46	21 (16–27)	
HGSC	29	20 (11–NA)	0.915
non-HGSC	22	22 (11–31)	
CR	25	23 (12–NA)	0.273
PR	26	19 (8–24)	
Elevated CA125 (>35)	37	22 (12–31)	0.928
Normal CA125	14	20 (8–NA)	
Elevated CRP (>0.1)	30	20 (11–NA)	0.787
Normal CRP	21	22 (11–NA)	
Elevated LDH (>222)	13	31 (5–NA)	0.163
Normal LDH	38	20 (11–24)	
Elevated SII (>730)	9	11 (5–NA)	0.207
Normal SII	41	22 (18–31)	
Elevated NLPN score (>7.51)	8	13.5 (5–NA)	0.279
Normal NLPN score	42	22 (19–31)	
Elevated NLR (>3)	9	11 (5–NA)	0.235
Normal NLR	41	22 (18–31)	
Ola	34	27 (19–NA)	0.000103
Bev	17	9 (5–22)	

m, mutation; wt, wild type; HGSC, high-grade serous carcinoma; HRD, homologous recombination deficient; CR, complete response; PR, partial response; CA, cancer antigen; LDH, lactate dehydrogenase; CRP, C-reactive protein; NLPN score, neutrophil–lymphocyte ratio at recurrence × previous number of regimens; NLR, neutrophil–lymphocyte ratio; SII, systemic inflammatory index; Ola, olaparib; Bev, bevacizumab.

## Data Availability

Datasets are available from the corresponding author upon reasonable request.

## References

[1] Thigpen J.T., Blessing J.A., Ball H., Hummel S.J., Barrett R.J. (1994). Phase II trial of paclitaxel in patients with progressive ovarian carcinoma after platinum-based chemotherapy: A Gynecologic Oncology Group study. J. Clin. Oncol..

[2] NCCN (2022). Clinical Practice Guidelines in Oncology (NCCN Guidelines®) Ovarian Cancer Including Fallopian Tube Cancer and Primary Peritoneal Cancer. Version 1.2023. https://www.nccn.org/professionals/physician_gls/pdf/ovarian.pdf.

[3] Tokunaga H., Mikami M., Nagase S., Kobayashi Y., Tabata T., Kaneuchi M., Satoh T., Hirashima Y., Matsumura N., Yokoyama Y. (2021). The 2020 Japan Society of Gynecologic Oncology guidelines for the treatment of ovarian cancer, fallopian tube cancer, and primary peritoneal cancer. J. Gynecol. Oncol..

[4] Nakanishi K., Yamada T., Ishikawa G., Suzuki S. (2021). Beyond BRCA status: Clinical biomarkers may predict therapeutic effects of olaparib in platinum-sensitive ovarian cancer recurrence. Front. Oncol..

[5] Nguyen J.M.V., Ferguson S.E., Bernardini M.Q., May T., Laframboise S., Hogen L., Bouchard-Fortier G. (2020). Preoperative neutrophil-to-lymphocyte ratio predicts 30 day postoperative morbidity and survival after primary surgery for ovarian cancer. Int. J. Gynecol. Cancer.

[6] Yin X., Wu L., Yang H., Yang H. (2019). Prognostic significance of neutrophil-lymphocyte ratio (NLR) in patients with ovarian cancer: A systematic review and meta-analysis. Medicine.

[7] Nie D., Gong H., Mao X., Li Z. (2019). Systemic immune-inflammation index predicts prognosis in patients with epithelial ovarian cancer: A retrospective study. Gynecol. Oncol..

[8] Kanda Y. (2013). Investigation of the freely available easy-to-use software “EZR” for Medical Statistics. Bone Marrow Transplant..

[9] Eisenhauer E.A., Therasse P., Bogaerts J., Schwartz L.H., Sargent D., Ford R., Dancey J., Arbuck S., Gwyther S., Mooney M. (2009). New response evaluation criteria in solid tumours: Revised RECIST guideline (version 1.1). Eur. J. Cancer.

[10] Aghajanian C., Blank S.V., Goff B.A., Judson P.L., Teneriello M.G., Husain A., Sovak M.A., Yi J., Nycum L.R. (2012). OCEANS: A randomized, double-blind, placebo-controlled phase III trial of chemotherapy with or without bevacizumab in patients with platinum-sensitive recurrent epithelial ovarian, primary peritoneal, or fallopian tube cancer. J. Clin. Oncol..

[11] Coleman R.L., Brady M.F., Herzog T.J., Sabbatini P., Armstrong D.K., Walker J.L., Kim B., Fujiwara K., Tewari K.S., O’Malley D.M. (2017). Bevacizumab and paclitaxel–carboplatin chemotherapy and secondary cytoreduction in recurrent, platinum-sensitive ovarian cancer (NRG Oncology/Gynecologic Oncology Group study GOG-0213): A multicentre, open-label, randomised, phase 3 trial. Lancet Oncol..

[12] Ledermann J., Harter P., Gourley C., Friedlander M., Vergote I., Rustin G., Scott C., Meier W., Shapira-Frommer R., Safra T. (2012). Olaparib maintenance therapy in platinum-sensitive relapsed ovarian cancer. N. Engl. J. Med..

[13] Pujade-Lauraine E., Ledermann J.A., Selle F., Gebski V., Penson R.T., Oza A.M., Korach J., Huzarski T., Poveda A., Pignata S. (2017). Olaparib tablets as maintenance therapy in patients with platinum-sensitive, relapsed ovarian cancer and a BRCA1/2 mutation (SOLO2/ENGOT-Ov21): A double-blind, randomised, placebo-controlled, phase 3 trial. Lancet Oncol..

[14] Mirza M.R., Monk B.J., Herrstedt J., Oza A.M., Mahner S., Redondo A., Fabbro M., Ledermann J.A., Lorusso D., Vergote I. (2016). Niraparib maintenance therapy in platinum-sensitive, recurrent ovarian cancer. N. Engl. J. Med..

[15] Ray-Coquard I., Mirza M.R., Pignata S., Walther A., Romero I., du Bois A. (2020). Therapeutic options following second-line platinum-based chemotherapy in patients with recurrent ovarian cancer: Comparison of active surveillance and maintenance treatment. Cancer Treat. Rev..

[16] Lee C.K., Friedlander M.L., Tjokrowidjaja A., Ledermann J.A., Coleman R.L., Mirza M.R., Matulonis U.A., Pujade-Lauraine E., Bloomfield R., Goble S. (2021). Molecular and clinical predictors of improvement in progression-free survival with maintenance PARP inhibitor therapy in women with platinum-sensitive, recurrent ovarian cancer: A meta-analysis. Cancer.

[17] Kim S.I., Lee J.W., Kim K., Lee M., Yoo J., Choi M.C., Hwangbo S., Kwak Y.H., Lee J.M., Shin S.J. (2021). Comparisons of survival outcomes between bevacizumab and olaparib in BRCA-mutated, platinum-sensitive relapsed ovarian cancer: A Korean Gynecologic Oncology Group study (KGOG 3052). J. Gynecol. Oncol..

[18] Yoshihama T., Kuroda Y., Chiyoda T., Takahashi M., Yoshimura T., Saotome K., Nanki Y., Sakai K., Kobayashi Y., Yamagami W. (2022). Efficacy and safety of olaparib maintenance monotherapy for Japanese patients with platinum-sensitive relapsed ovarian, fallopian tube, and primary peritoneal cancer. Int. J. Clin. Oncol..

[19] Gao Q., Zhu J., Zhao W., Huang Y., An R., Zheng H., Qu P., Wang L., Zhou Q., Wang D. (2022). Olaparib maintenance monotherapy in Asian patients with platinum-sensitive relapsed ovarian cancer: Phase III trial (L-MOCA). Clin. Cancer Res..

[20] Musella A., Vertechy L., Romito A., Marchetti C., Giannini A., Sciuga V., Bracchi C., Tomao F., Di Donato V., De Felice F. (2017). Bevacizumab in ovarian cancer: State of the art and unanswered questions. Chemotherapy.

